# Pixelwise high-temperature calibration for in-situ temperature measuring in powder bed fusion of metal with laser beam

**DOI:** 10.1016/j.heliyon.2024.e28989

**Published:** 2024-04-02

**Authors:** Dennis Höfflin, Christian Sauer, Andreas Schiffler, Jochen Manara, Jürgen Hartmann

**Affiliations:** a Technical University of Applied Sciences Würzburg-Schweinfurt, Germany; b Center for Applied Energy Research e.V. (CAE), Würzburg, Germany

**Keywords:** Process monitoring, High-temperature calibration, PBF-LB/M

## Abstract

High-temperature calibration methods in additive manufacturing involve the use of advanced techniques to accurately measure and control the temperature of the build material during the additive manufacturing process. Infrared cameras, blackbody radiation sources and non-linear optimization algorithms are used to correlate the temperature of the material with its emitted thermal radiation. This is essential for ensuring the quality and repeatability of the final product. This paper presents the calibration procedure of an imaging system for in-situ measurement of absolute temperatures and temperature gradients during powder bed fusion of metal with laser beam (PBF-LB/M) in the temperature range of 500 K–1500 K. It describes the design of the optical setup to meet specific requirements in this application area as well as the procedure for accounting the various factors influencing the temperature measurement. These include camera-specific effects such as varying spectral sensitivities of the individual pixels of the sensor as well as influences of the exposure time and the exposed sensor area. Furthermore, influences caused by the complex optical path, such as inhomogeneous transmission properties of the galvanometer scanner as well as angle-dependent transmission properties of the f-theta lens were considered. A two-step fitting algorithm based on Planck's law of radiation was applied to best represent the correlation. With the presented procedure the calibrated thermography system provides the ability to measure absolute temperatures under real process conditions with high accuracy.

## Graphical abstract

**Figure:**
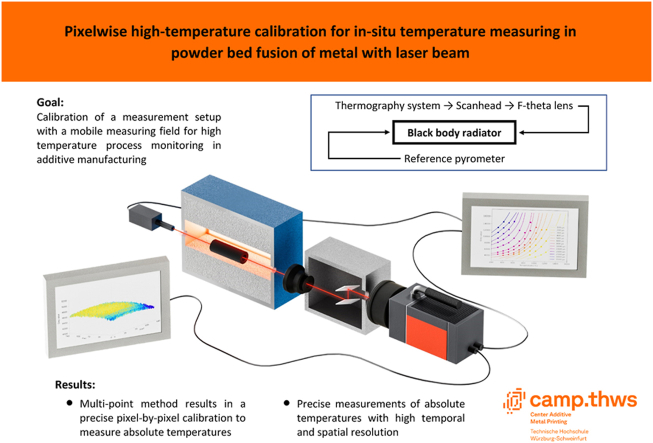

## 1 Introduction

Metal additive manufacturing (AM) is a process of creating complex 3D objects by building up layers of material. It is a rapidly growing process technology with a wide range of applications, including aerospace [1], biomedical [2] and automotive industries [3]. Temperature measurement is a critical aspect of the AM process, as it plays a vital role in ensuring the quality, repeatability and reliability of the final product [4]. Overheating can lead to balling effects and other defects that affect the process stability and the quality of the component. Falling below a certain temperature in the process zone can lead to lack-of-fusion defects. The high melting and cooling rates of the material induce thermal stress, which can cause thermal distortion and deformation if not properly controlled. To address these issues, the trend is moving from process data monitoring [5] over closed-loop process control [6,7] to real-time adjustments [8] of the applied process parameters.

Various existing approaches use sensors to derive indicators for process assessment from the thermal process signature and thus make in-situ statements about process stability, component quality or expected properties. Monitoring systems for PBF-LB/M detect geometric deviations from the target contour based on the thermal signature and/or detect process anomalies such as spattering and powder spreading issues [9,10].

## 2 Methods

### 2.1 SPIT setup

**Fig. 1:**
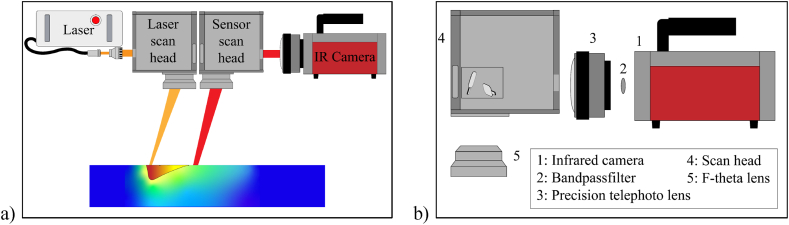
a) schematic representation of the SPIT setup b) exploded view of the sensor path and its optical components.

The thermography system used and calibrated in this investigation is the one that is used in the SPIT approach, basically shown in Fig. 1a [5]. The SPIT setup is originally installed in a customised research platform designed to recreate the PBF-LB/M process in a simplified way and to analyze the thermal processes in detail. It is used to determine temperatures during the cooling process in an area apart from the laser-material interaction zone in which critical factors, such as different aggregate states or welding fumes, do not influence the measurement. The key here are two parallel arranged scan heads that can be operated separately from each other. The laser scan head (SCANLAB intelliSCAN III 20; 1055–1085 + 880 nm) including an f-theta lens (SCANLAB solid quarz lens; f = 420-1064-4) is utilised to guide the processing laser (VONJAN Technology GmbH, single mode fiber laser CW 500 SM, wavelength 1080 nm) over the built platform. The sensor scan head (SCANLAB intelliSCAN III 20; 450–2500 nm) including an f-theta lens (SCANLAB solid quarz lens; f = 437-[1940–2050]-1) is designed for longer wavelength infrared radiation in order to steer the measurement field over the platform.

In the sensor unit, the thermal process radiation during the cooling process is guided through the optical path of the scan head onto the sensor of an infrared camera (InfraTec IR 8300). The optical paths consists of a f-theta lens, the deflection mirrors of the scan head, the telephoto lens of the IR camera as well as a narrow bandpass filter (bpf), that is optional depending on the measurement task. The optical path of this system is optimized for a wavelength range from 2000 nm to 2050 nm. The use of the bandpass filter further narrows the useable range to 2040 nm ± 8 nm. The relevant components of the setup are shown in Fig. 1b.

Because the system aims to detect the temperatures during material cooling, the sensitive wavelength range is adapted accordingly to the expected temperatures. The sensitivity ranges of the individual components are listed in Table 1. Another application of the system is to include in-situ material examinations by active thermography. Here, the material is thermally excited by the processing laser and the heat propagation is detected by the IR camera. The heating takes place in a temperature range far below the melting point. For this purpose, observing the material with a broader wavelength range is necessary to ensure a sufficient signal-to-noise ratio at low temperatures. Therefore, the calibration was carried out for different optical path configurations. A low-temperature range configuration without the bandpass filter (see Fig. 1b), as well as a high-temperature range configuration with the built-in bandpass filter.

**Table 1:** *Overview of the optical elements used in the sensor path and their supporting wavelength range (minimum wavelength λ*_min_*and maximum wavelength λ*_max_).

Optical element	$\lambda_{\min}$ [nm]	$\lambda_{\max}$ [nm]
Sensor (IR-camera)	1500	5100
Bandpass filter	2032	2048
Telephoto lens	2000	5500
Mirrors (Scan head)	450	2500
F-theta lens	1940	2050

### 2.2 Calibration setup

**Fig. 2:**
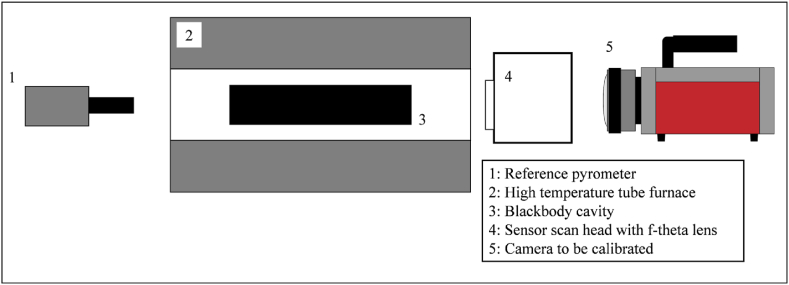
Schematic representation of the calibration setup.

A high-temperature calibration setup was used to calibrate the sensor system including the entire optical path to establish a radiometric model for reliable in process temperature measurements. The setup consists of a reference pyrometer to measure the calibration temperature (HEITRONICS KT15.43 IIP), a high-temperature tube furnace (Gero HTRH 70–600/18) with a temperature resistant ceramic tube, a blackbody cavity and the sensor system that is used in the SPIT setup shown in Fig. 1b. The schematic arrangement is shown in Fig. 2.

The blackbody cavity was placed in the center of the ceramic tube, where the oven ensures homogeneous temperature distribution. The pyrometer was then positioned in front of the furnace and focused on the aperture of the cavity with a measuring field much smaller than the aperture. At the same time, the infrared camera and optical path were placed on the opposite side of the furnace, with the same working distance between f-theta lens and the front of the cavity as in the SPIT setup. This ensures that the sensor area directed to the opening of the cavity was more than 70% of the sensor area.

### 2.3 Blackbody cavity

**Fig. 3:**
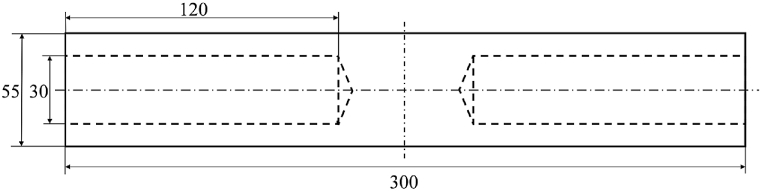
Technical drawing of the graphite cavity. Dimensions are given in mm.

To enable simultaneous measurements with the reference pyrometer as well as with the camera under calibration, a double-sided blackbody cavity was used (cf. Fig. 3). The cylindrical shaped cavity was made of graphite with holes on both sides. For high emissivity values the holes had inclined bottoms and a large depth to diameter ratio. A simple method for estimating the overall emissivity of the cavity radiator, $\varepsilon_{\text{cavity}}$, with the base material emissivity $\varepsilon_{\text{graphite}}$, length l and radius r was given by Bauer and Bischoff in 1970 [17] as

(1) $$\varepsilon_{\text{cavity}}=1-\frac{1-\varepsilon_{\text{graphite}}}{\varepsilon_{\text{graphite}}}\frac{1}{1+\left(\frac{l^{2}}{r^{2}}\right)}$$

The emissivity of the graphite as base material was previously determined in separate measurements [18]. An emissivity of $\varepsilon_{\text{graphite}}=$ 0.85 at a wavelength of 2 μm was determined for the relevant temperature range. Therefore, an overall emissivity of the blackbody cavity was calculated with Eq. (1) as $\varepsilon_{\text{cavity}\;}$ = 0,997.

### 2.4 Calibration procedure

#### 2.4.1 Theory

The relation between the spectral infrared radiation $L_{\text{bb}}$ and the temperature $T_{\text{bb}}$ of a blackbody as a function of wavelength λ is given by Planck's law [19]:

(2) $$L_{\text{bb}}\left(\lambda ,T_{\text{bb}}\right)=\frac{c_{1}}{\lambda^{5}}\frac{1}{e^{\frac{c_{2}}{\lambda T_{\text{bb}}}}-1}$$

where $c_{1}$ and $c_{2}$ are physical constants [19]. Real surfaces are typically not equivalent to a blackbody. Therefore, the radiation emitted by real surfaces, $L_{\text{real}}$, is calculated by

(3) $$L_{\text{real}}\left(\lambda ,T_{\text{real}}\right)=\varepsilon \left(\lambda ,T_{\text{real}}\right)L_{\text{bb}}\left(\lambda ,T_{\text{bb}}\right)$$

where $\varepsilon \left(\lambda ,T_{\text{real}}\right)$ is the emissivity of the radiating surface with the actual temperature $T_{\text{real}}$. If the spectral irradiance is measured and the emissivity is known, the corresponding surface temperature can be calculated by using Eq. (2) and Eq. (3) with

(4) $$T_{\text{bb}}=\frac{c_{2}}{\lambda }\frac{1}{\ln\left(\frac{\varepsilon \left(\lambda \right)\times c_{1}}{L_{\text{bb}}\left(\lambda \right)\times \lambda^{5}}+1\right)}$$

#### 2.4.2 Characteristics of the infrared camera

The imaging system used in the present investigation is an InfraTec IR 8300 infrared camera with an indium antimonide sensor, which is sensitive in the wavelength range from 1 μm to 5 μm [MWIR]. The thermal radiation from the surface is focused through mirrors and lenses on the detector, which generates a corresponding photocurrent. The measured photocurrent of each pixel is then assigned a grey value (GV) in the range from 0 to $2^{14}-1$ that can be used to calculate the temperature of an object. The photocurrent $I_{\text{Photo}}$ can be written as

(5) $$I_{\text{photo}}=G\int_{\lambda =0}^{\infty }s\left(\lambda \right)\varepsilon \left(T_{\text{real}},\lambda \right)L_{\text{bb}}\left(T_{\text{bb}},\lambda \right)\;d\lambda$$

where G is the geometric factor and s(λ) is the spectral sensitivity of the sensor, i.e., the sensitivity in the resulting wavelength range including the effect of the optical path. For narrowband measurements at an effective wavelength $\lambda_{\text{eff}}$, which is given in this investigation through the limiting optical elements (see section 2.1) and by using Eq. (5), the resulting photocurrent (Eq. (6))

(6) $$I_{\text{photo}}=G\times s\left(\lambda_{\text{eff}}\right)\varepsilon \left(T_{\text{real}},\lambda_{\text{eff}}\right)L_{\text{bb}}\left(T_{\text{bb}},\lambda_{\text{eff}}\right)\Delta \lambda$$

can be expressed by the following simplified function [19]:

(7) $$GV=\frac{C}{e^{\frac{c_{2}}{A\;T-B}}-1}$$

The structure of Eq. (7) appears to be similar to Eq. (2) with A,B and C as parameters, which have to be determined to obtain the “best-fit” relation between measured and reference temperature. For small wavelengths and considerably low temperatures, Wien's formular can be used as approximation of Planck's law with sufficient accuracy. Thus, the measured grey values are fitted as a function of the temperature of the reference emitter with the following adapted fit function

(8) $$GV=C\;e^{\frac{{-c}_{2}}{A\;T_{\text{bb}}-B}}$$

Finally, by using Eq. (8), the temperature as a function of the grey value can therefore be expressed by the following term:

(9) $$T_{\text{bb}}=\frac{1}{A}\left(B-\frac{c_{2}}{\ln\left(\frac{GV}{C}\right)}\right)$$

To perform accurate measurements on real surfaces, it is necessary to consider the emissivity of the surface. When using spectral radiation thermometry under the condition that the ambient temperature $T_{a}\ll T_{\text{bb}},T_{\text{real}}$, the impact of emissivity on the measured temperatures can be expressed via the simplified relationship in Eq. (10) [20].

(10) $$\frac{1}{T_{\text{real}}}=\frac{1}{T_{\text{bb}}}+\frac{\lambda_{\text{eff}}}{c_{2}}\ln\;\varepsilon$$

Here, $T_{\text{real}}$ is the real temperature of a surface with emissivity $\varepsilon ,T_{\text{bb}}$ is the blackbody temperature and $\lambda_{\text{eff}}$ is the effective observation wavelength.

#### 2.4.3 Recording procedure

In the following, the calibration procedure with the previously presented setup is explained in detail. The test series initially aimed to determine optimal measurement settings for different scenarios (cooling behavior of the process, active thermography). Therefore, data was generated with different camera configurations (with/without bandpass filter, different sensor areas, various integration times).

One focus was laid on the integration time, which aimed to cover a wide temperature range with a single integration time. A grey value of 3 × 10³ was initially set as the lower limit, corresponding to the background noise of the setup. The upper limit was set to a grey value of 16 × 10³, above which the single pixel is saturated. The reason for the different sensor areas, respectively the number of evaluated pixels on the sensor, is a compromise between a large field of view and a high frame rate. For the observation of the cooling of the material behind the laser spot, a rather small viewing area at a high frame rate is necessary; for active thermography, a large viewing area at a medium frame rate is prioritized. In principle, small sensor areas allow higher framerates. Therefore, sensor areas of 140 × 40 and 140 × 4 pixels were examined for the optical path including the bandpass filter and sensor areas of 140 × 140 and 140 × 40 pixels for the setup without the bandpass filter. The center of the image section was always in the middle of the sensor area. An overview of the investigated integration time-temperature combinations is given in Table 2. As the calibration procedure was performed independently for each pixel, the raw data was the only input for the curve fitting, which did not involve any post-processing steps from the camera manufacturer such as bad pixel repair (BPR) or non-uniformity correction (NUC).

**Table 2:** Overview of the different integration time – temperature combinations, where data were generated (sensor area 140 × 40 pixels). The ‘x’ represents measurements without the bandpass filter, the ‘o’ marks measurements including the bandpass filter. For the 140 × 4 sensor area only parameter combinations with the ‘o’, for the 160 × 160 sensor area only parameter combinations with the ‘x’ were recorded.

		Temperature [°C]															
		180	220	250	270	320	370	420	470	520	620	720	820	920	1020	1120	1220
**Integration time [μs]**	25				x	x	x	x	x	x							
	50			x	x	x	x	x	x	x						o	o
	100		x	x	x	x	x	x	x	x					o	o	o
	200		x	x	x	x	x	x	x	x				o	o	o	o
	300	x	x	x	x	x	x	x	x	x				o	o	o	o
	400	x	x	x	x	x	x	x	x	x		o	o	o	o	o	o
	500	x	x	x	x	x	x	x	x	x	o	o	o	o	o	o	o
	600	x	x	x	x	x	x	xo	x	o	o	o	o	o	o	o	o
	800	x	x	x	x	xo	x	xo	x	o	o	o	o	o	o	o	
	1000	x	x	x	x	xo	x	xo	x	o	o	o	o	o	o		
	1500	x	x	x	x	xo	x	o		o	o	o	o	o	o		
	2000	x	x	x	x	xo		o		o	o	o	o	o			
	2500	x	x	x		o		o		o	o	o					
	3000	x	x			o		o		o	o						
	3500	x				o		o		o	o						

For each camera configuration, the cavity was gradually heated to defined temperatures and the reference pyrometer as well as the camera measured simultaneously from both sides under steady-state conditions. The pyrometer measured the exact temperature, while the camera under calibration recorded a set of 20 images at 10 fps. The oven temperature was assumed to be constant during these 2 s. During thermal image recording all user-defined parameters (e.g., temperatures of the atmosphere and the surroundings, humidity of the atmosphere etc.) were recorded.

#### 2.4.4 Curve fitting procedure

**Fig. 4:**
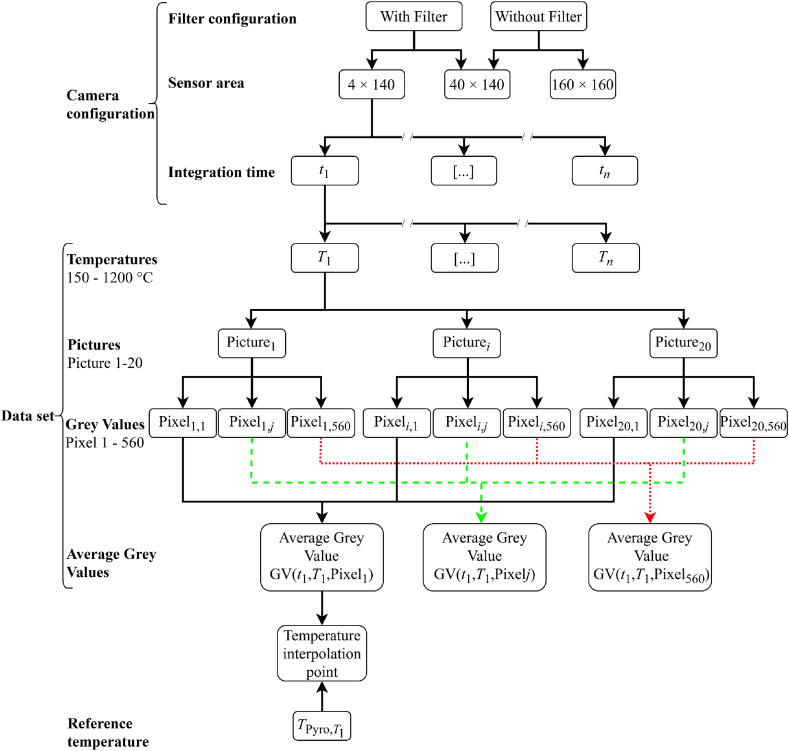
Overview of the procedure for generating calibration data. The procedure is shown as an example for the camera configuration with filter, sensor area 140 × 4 pixels, integration time t _1_ and was implemented analog for all configurations.

As stated above, the fitting procedure is based on the pyrometer values and the corresponding thermal images recorded concurrently under steady-state test conditions to find the parameters A,B,C of Eq. (9) and minimize the rms error. Calibration of each sensor pixel was done independently, allowing individual compensation for sensitivity deviations for each pixel. The measured reference temperatures and the associated grey values were used as interpolation points for the fit using Eq. (9). Given the assumption of normal distribution for the random error (image noise) of the IR camera, the N images captured at each reference temperature were aggregated by computing their mean value (pixelwise). The primary objective of this was to mitigate the bias in the final output by a factor of N. Depending on the camera configuration, the total number of interpolation points in the range between the lower and upper grey value limit varied. The entire procedure is shown schematically in Fig. 4.

To fit the data to the base function (Eq. (9)), a curve-fit function in Python with the method parameter set to ‘dogbox’ was used. Dogbox is based on the dogleg algorithm which is a variant of the Levenberg-Marquardt algorithm using rectangular trust regions [21]. It is designed to handle situations where the model has a wide range of parameter values. Overall, the dogbox algorithm provides a robust and accurate method for fitting the data to the model function, even in situations where other optimization methods struggle.

#### 2.4.5 Position dependence

**Fig. 5:**
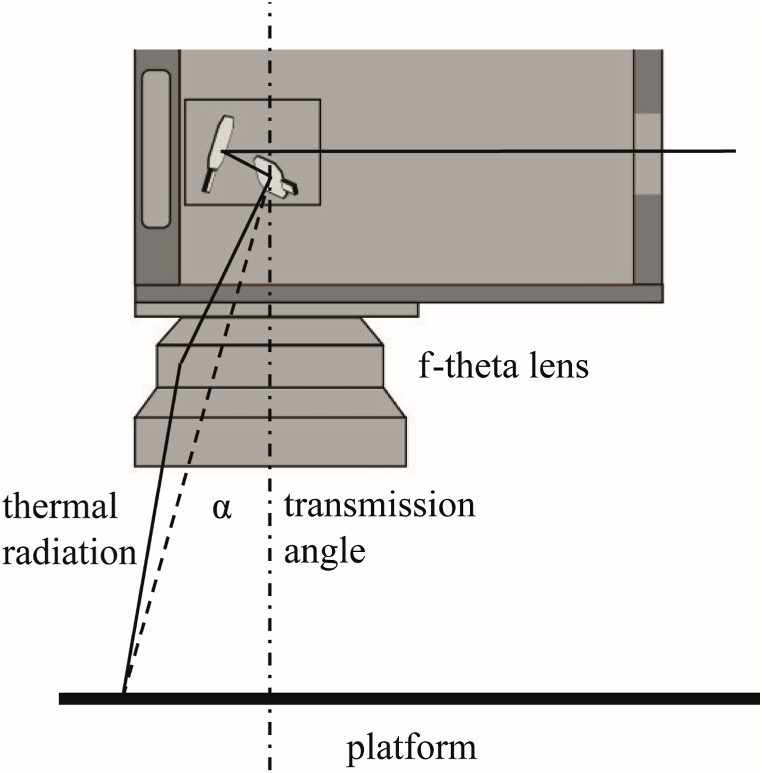
Simplified representation of the transmission angle α of the thermal radiation through the f-theta lens.

During the basic calibration process, the characteristic of the process radiation passing the f-theta lens was measured in vertical direction (with an angle of α=0, see Fig. 5). To determine the degree to which the f-theta lens transmits radiation at various transmission angles, the optical components of the setup (scan head with the f-theta lens and camera) were incrementally displaced while maintaining a constant furnace temperature. The camera was focused on the cavity during these movements. The step size was 10 mm in a range of 130 mm from the center, resulting in an absolute transmission angle α of 13.8°. The measuring point of the camera remained stationary on the cavity opening. Owing to the lens's rotational symmetry, these measurements were performed in one axis only and extrapolated to the other regions of the working surface. The supplementary position-dependent attenuation factor obtained in this manner is included in the calibration as an additional factor.

### 2.5 Surface emissivity

Because the intention of the SPIT setup is to measure absolute temperatures in a region behind the laser-material interaction zone and therefore after re-solidification, it is possible to determine the occurring emissivity values for the material of interest in prior measurements. In order to determine the properties of the as printed surface, emissivity measurements of stainless steel (1.4540) samples, produced on an EOS M290 PBF-LB/M machine, were carried out. To ensure comparable properties in terms of material and surfaces, separate parameters for the production of the upskin were not applied. The emissivity of the resolidified material was measured in a total of eight different surface temperatures, ranging from 400 °C to 1240 °C at a center wavelength of 2040 nm. This was carried out with an apparatus, specially designed for measuring the emissivity at high temperatues [22]. Due to the narrowband measurements the emissivity was assumed to be constant over the investigated wavelength range. The resulting emissivity curve was integrated into the calculations using Eq. (4), enabling the correction of raw temperature data for emissivity effects. The core of the methodology involved an iterative refinement process. In each iteration, the temperature adjustment resulting from emissivity corrections was recalculated, thereby progressively narrowing the discrepancy in the temperature readings. This iterative cycle was repeated until the difference in the corrected temperature fell below a threshold of 0.01 K, ensuring minimal error due to emissivity variations.

### 2.6 Uncertainty analysis

The uncertainty analysis accounted for the influences of the different components involved in the calibration process as well as additional factors (e.g., pyrometer uncertainty). The uncertainty of the reference pyrometer provided by Heitronics was directly obtained from the manufacturer's specifications. To determine the level of uncertainty associated with the infrared thermography system itself, a statistical analysis was conducted to evaluate the degree of measurement noise. This analysis involved calculating the standard deviation V of the data obtained by the system. Therefore Eq. (11) was used by considering data from 20 separate measurements:

(11) $$V=\sqrt{\frac{1}{n-1}\sum_{i=1}^{n}{\left[T_{\text{bb},i}-\left(\frac{1}{n}\sum_{i=1}^{n}T_{\text{bb},i}\right)\right]}^{2}}$$

The mean statistical uncertainty $u_{r,\bar{T}}$ from the true value due to statistical variation was determined by applying Student's t-distribution with a 95% confidence interval (Eq. (12)).

(12) $$u_{r,\bar{T}}=t_{n,95}\;V$$

To calculate the overall temperature uncertainty $u_{temp}$, the uncertainties of every individual influencing factor $u_{x_{i}}$ are subsequently incorporated into the calculation of uncertainty propagation, including their sensitivity factors, which can be written as

(13) $$u_{temp}=\sqrt{\sum_{i=1}^{n}u_{x_{i}}^{2}{\left(\frac{\partial T_{real}}{\partial x_{i}}\right)}^{2}}$$

## 3 Results

**Fig. 6:**
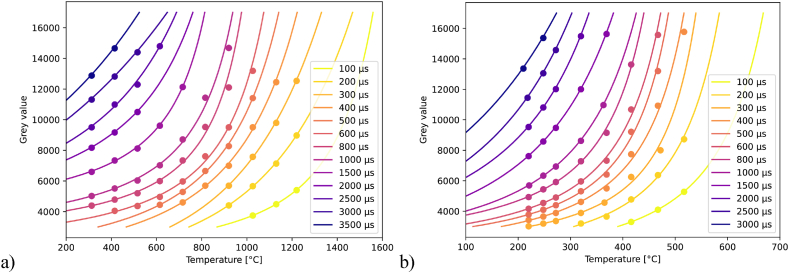
Overview of the measuring points of the individual parameter sets for a) setup including the bpf, b) setup without the bpf. For simplified representation, each point corresponds to the mean value of all pixels of the 20 images taken at one temperature. To further improve the overview, the fit function was applied to these values in each case.

Applying the procedures described above, the thermographic data was collected and processed. The measurements and evaluations were carried out identically for the different sensor areas. The results of the statistical evaluation differ only marginally. For the sake of convenience, only the results of the measurement series for the sensor area of 140 × 40 pixels are presented below in detail. The courses of the raw data are shown in Fig. 6 (a,b) in simplified form. Here, every interpolation point represents the average grey value of the 20 images taken at one temperature. The results indicate that optimal integration times vary depending on the intended applications. The calibration data were chosen based on the camera setting that maximized the thermal resolution for subsequent analyses. Moreover, the achievable framerates at these integration times were considered, which also influenced the calibration of different image section dimensions.

### 3.1 Operation modes

**Fig. 7:**
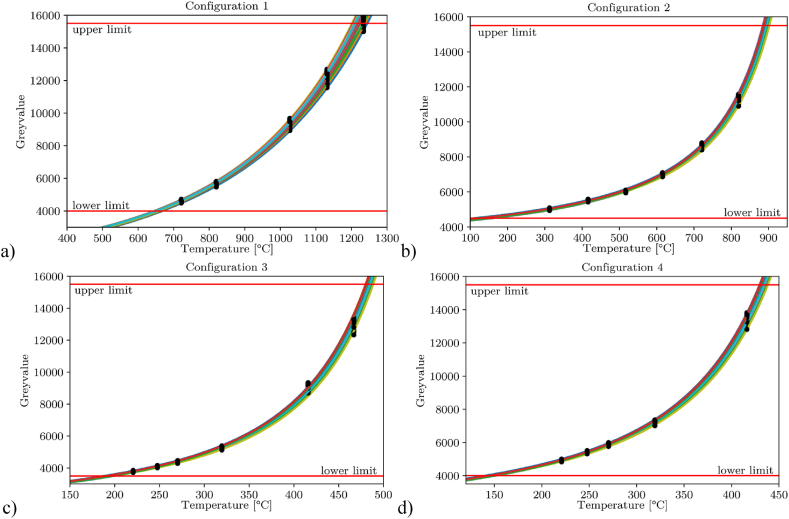
Plot of the grey values over temperature for every of the 140 × 40 pixel including the corresponding fitting curves with a) Configuration 1, with bpf and 400 μs integration time; b) Configuration 2, with bpf and 1000 μs integration time; c) Configuration 3, without bpf and 500 μs integration time; d) Configuration 4, without bpf and 800 μs integration time.

Based on the results of Fig. 6, four different parameter configurations were chosen for the further calibration process. As stated above, the main criteria were a broad observable temperature range and a good signal-to-noise ratio. Therefore, integration times of 400 μs and 1000 μs were selected for the high-temperature range including the bandpass filter, and 500 μs and 800 μs for the low temperature range without the filter. The measured data of the parameter sets are shown in Fig. 7(a–d) including the interpolation points and fitting curves for each of the 5600 pixels. The fluctuations between the pixels can be attributed to different sensitivities of the individual pixels of the sensor as well as the complex optical path and lie within a range of 6 % over the investigated temperature range. During the investigation, the upper threshold was lowered from a grey value of 16 × 10³ to 15.5 × 10³ as first saturation effects could be observed at this value. The lower threshold changed with the integration time and had to be chosen individually. The different configurations and the corresponding recording parameters are shown in Table 3.

**Table 3:** Selected camera configurations and the corresponding parameters.

	Config. 1	Config. 2	Config. 3	Config. 4
Integration time [μs]	400	1000	500	800
Bandpass filter	Yes	Yes	No	No
Minimum temperature [°C]	640	150	175	140
Maximum temperature [°C]	1220	890	485	430
Lower GV limit	4000	4500	3500	4000
Upper GV limit	15500	15500	15500	15500

The maximum achievable frame rates with the respective parameter settings are shown in Table 4. It is noticeable that for small sensor areas the integration time is the limiting factor, therefore no improvement could be achieved by reducing the sensor area.

**Table 4:** Overview of the maximum frames per second for the different camera configurations, depending on the sensor area.

Sensor area	Camera configuration			
	Config. 1	Config. 2	Config. 3	Config. 4
140 × 4	1904 fps	882 fps	–	–
140 × 40	1904 fps	882 fps	1592 fps	1065 fps
140 × 140	–	–	844 fps	850 fps

### 3.2 Correction of systemic error

**Fig. 8:**
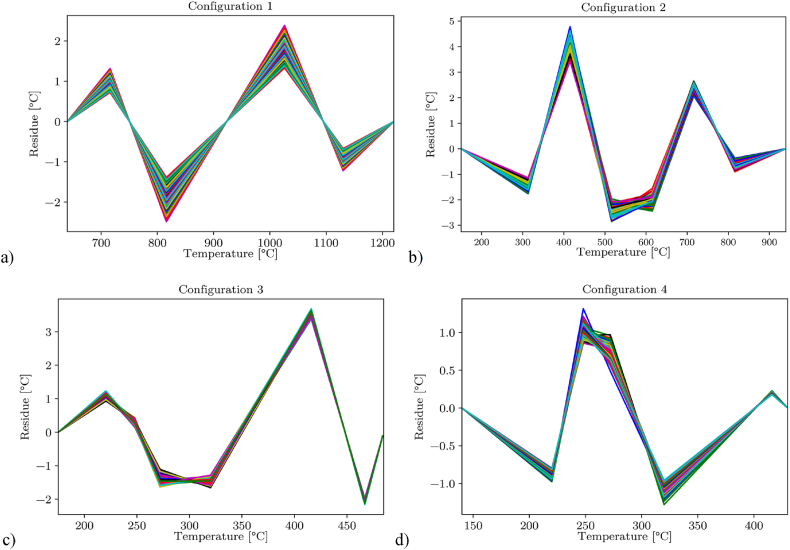
Representation of the curve fitting residuals over the temperature for every of the 140 × 40 pixel with a) Configuration 1, with bpf and 400 μs integration time; b) Configuration 2, with bpf and 1000 μs integration time; c) Configuration 3, without bpf and 500 μs integration time; d) Configuration 4, without bpf and 800 μs integration time.

To evaluate the characteristics of the curve fit, the residuals at the interpolation points were examined in detail. The deviations between the interpolation points and the fitted curve were similar for all pixels. The residuals of all pixels over the temperature are shown in Fig. 8(a–d), presented for each camera configuration, and were within a range of 0 °C–15 °C. To further enhance the precision of the overall calibration, the measured residuals were subsequently subtracted from the initial curve fit in a second correction step. The areas between the grid points were linearly interpolated. Thus, the correction was described with a piecewise function individually generated for each pixel depending on the residues of the interpolation points. The correction function is given by:

(14) $${\Delta T}_{res}\left(T\right)=\left\{ \begin{array}{c} a_{1}\;T_{\text{real}}+b_{1}\;\\ a_{n}{\;T}_{\text{real}}+b_{n}\;\\ a_{n+1}\;T_{\text{real}}+b_{n+1} \end{array}\right.\;\begin{array}{c} \text{for}\;T_{\text{real}}\in ]T_{\text{real},0},T_{\text{real},1}]\;\\ \text{for}\;T_{\text{real}}\in ]T_{\text{real},1},T_{\text{real},n}]\;\\ \text{for}\;T_{\text{real}}\in ]T_{\text{real},n},T_{\text{real},n+1}] \end{array}$$

Here, a is the slope of the section between two interpolation points. The final temperature then results from the difference of Eq. (9) and Eq. (14).

### 3.3 Geometrical dependency of transmission factor

**Fig. 9:**
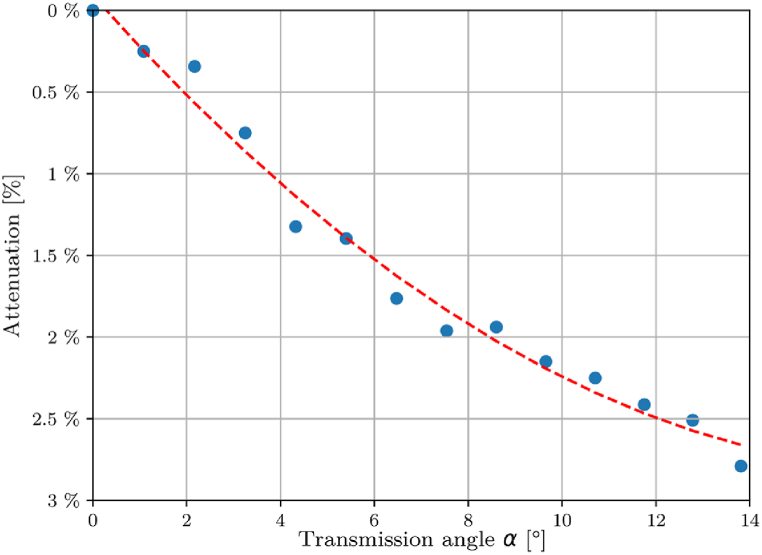
Plot of the angle-dependent attenuation arising from the propagation of radiation through the f-theta lens including the fit function.

The angle-dependent transmission factor of the f-theta lens is a key parameter for determining the accurate temperature distribution on the build platform. Fig. 9 presents the results of the measurements performed to evaluate this factor. The data indicate a significant reduction in the grey values with increasing angle. The maximum attenuation is 2.7 % at an transmission angle of 13.8°, see Fig. 5. For the calculation of absolute temperatures, the fitted curve is a second order polynomial and the angle dependent attenuation factor awas used to correct the determined grey values.

### 3.4 Uncertainty analysis of the calibration

As written in section 2.6 Uncertainty analysis, all influencing factors of the measurement were considered for the overall error propagation analysis. Expanding Eq. (13) results in the following equation:

(15) $$u_{temp}=\sqrt{{\left(\frac{\partial T}{\partial T_{\text{bb},\text{cam}}}u_{T_{\text{bb},\text{cam}}}\right)}^{2}+{\left(\frac{\partial T}{\partial \varphi_{\alpha }}u_{\varphi_{\alpha }}\right)}^{2}+{\left(\frac{\partial T}{\partial \varepsilon }u_{\varepsilon }\right)}^{2}+{\left(\frac{\partial T}{\partial {\Delta T}_{\text{cavity}}}u_{{\Delta T}_{\text{cavity}}}\right)}^{2}+{\left(u_{T_{\text{bb},\text{pyro}}}\right)}^{2}}$$

$T_{\text{bb}}$ represents the temperatures measured at the blackbody cavity. Minor deviations of the measured temperatures caused by discrepancies in the emissivity of the cavity where neglected. The correction factor $\varphi_{\alpha }$ denotes the angular deviation in transmissivity, *ε* represents the emissivity of the material and ${\Delta T}_{\text{cavity}}$ is the temperature difference of the two sides of the blackbody radiator. Additionally, u represents the uncertainties associated with the respective values. The uncertainty of the measured emissivity $u_{\varepsilon }$ was determined with a value of 0.5% within the scope of the measuring accuracy of the apparatus used. The emissivities resulting from the relevant temperature levels are in a range between 0.43 and 0.45 in a temperature range from 400 °C to 1200 °C. Using Eq. (15), the resulting uncertainty budgets for the different configurations are shown in Table 5.

**Table 5:** Overall uncertainty budgets of the different calibration configurations. The ε-values for the minimum and the maximum temperatures of the specific configurations were applied.

Config	Uncertainty budgets						
	$u_{T_{\text{bb},\text{pyro}}}$	$u_{\varphi_{\alpha }}$	$u_{T_{\text{bb},\text{cam}}}$	$u_{{\Delta T}_{\text{cavity}}}$	$u_{\varepsilon }$	Resulting uncertainty	min/max
1	± $\left(0,7{}^{\circ}C+0,7\%T_{\text{real}}\right)$	±0,26% $T_{\text{real}}$	±1,3 °C	±1 °C	±0,5%	±($0,36{}^{\circ}C+0,0090\times T_{\text{real}})$	**6°C – 11°C**
2	± $\left(0,7{}^{\circ}C+0,7\%T_{\text{real}}\right)$	±0,26% $T_{\text{real}}$	±3,5 °C	±1 °C	±0,5%	±($3,66{}^{\circ}C+0,0065{\times T}_{\text{real}})$	**5°C – 10°C**
2	± $\left(0,7{}^{\circ}C+0,7\%T_{\text{real}}\right)$	±0,26% $T_{\text{real}}$	±1,0 °C	±1 °C	±0,5%	±($1,36{}^{\circ}C+0,0071{\times T}_{\text{real}})$	**3°C – 5°C**
4	± $\left(0,7{}^{\circ}C+0,7{\%T}_{\text{real}}\right)$	±0,26% $T_{\text{real}}$	±0,8 °C	±1 °C	±0,5%	±($1,43{}^{\circ}C+0,007{\times T}_{\text{real}})$	**2°C – 4°C**

## 4 Discussion

The results in this study show the possibility of accurately calibrating an infrared camera including a complex optical path for dynamic high-speed measurements of absolute temperatures in additive manufacturing with high accuracy. A unique feature of this system is the adjustable measuring field, allowing to analyze the moving process zone with high spatial and temporal resolution at various locations within the built platform.

In contrast to the 3-point calibration commonly used in industry [23], a multi-point calibration with up to 6 interpolation points per parameter set was chosen. The resulting correlations between grey values and actual temperatures thus consider the wavelength-dependent transmission properties of the complex optical path over the entire temperature range.

As simplification of Planck's radiation law (see Eq. (2)) Wien's law was used in this work for curve fitting (cf. Eq. (9)), which describes the physical relation between emitted thermal radiation of an ideal radiator and its temperature for short wavelengths and considerable low temperatures with sufficient accuracy. This was assured by the narrow bandwidth of the measurements, which ensures a nearly temperature independent effective wavelength $\lambda_{\text{eff}}$. Further, Wien's approximation shows good agreement with Planck's law under the condition that $\lambda_{\text{eff}}\;T_{\text{real}}<2897.8\;\mu \text{mK}$ [19,20]. With the effective wavelength $\lambda_{\text{eff}}$ of the measurements being 2040 nm, this condition is met for temperatures up to 1420 K. Due to the characteristics of the investigated process and the correspondingly relevant temperature ranges of the solidified material, in this case the melting point of stainless steel 1.4540, this condition is always fulfilled.

**Fig. 10:**
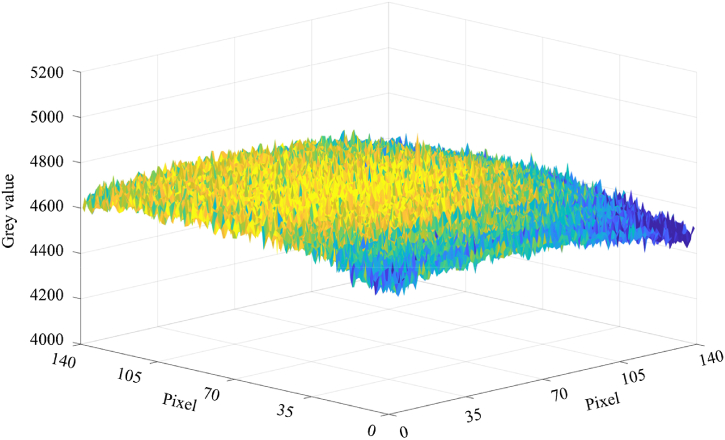
Representation of the raw grey value distribution over the exposed sensor area. The number of pixels is 140 × 140.

Due to the intricate optical pathway of the thermography system, it is crucial to carry out an elaborate calibration process on a pixel-by-pixel basis. This is necessary to account for both the distinct sensitivities of individual pixels of the camera and the non-uniform radiation intensity across the sensor area. The need for pixelwise calibration can be seen by the distribution of raw grey values for each pixel on the sensor surface, illustrated in Fig. 10. This snapshot was taken during the calibration procedure, where the entire exposed sensor area was subjected to the same temperature. Apart from the inherent pixel noise, the lower grey values at the edges of the sensor surface indicate the presence of various optical effects. These effects become more pronounced as the sensor area increases.

According to Minkina [24], the process conditions with greatest influence on measurement accuracy in infrared thermography are the material emissivity *ε*, the ambient temperature $T_{a}$, the atmospheric temperature ${\;T}_{\text{atm}}$, the relative humidity ω and the camera-to-object distance d. Given the specific experimental setup and the implemented calibration procedure, these assumptions have slightly shifted. The impact of the atmospheric temperature, the humidity, and the camera-to-object distance were disregarded in the uncertainty calculations. This was due to the calibration process being performed under constant conditions akin to those during actual measurements. Moreover, the narrow spectral bandwidth and the short camera-to-object distance (d = 810 mm) permit the negligence of attenuation caused by atmospheric components as those are not significant in relation to the other measurement uncertainties [25]. The impact of ambient temperature on the uncertainty calculations was neglected due to the substantial difference between the high process temperature ($T_{\text{process}}$) and the low ambient temperature ($T_{a}$). Hence, any minor heating of the surroundings is not expected to cause significant effects. Additionally, the parts of the optical path, which are directly exposed to thermal radiation and could potentially alter the measurements by causing distortion (primarily the scan head), are maintained at a consistent operating temperature and were already accounted for in the calibration measurements.

In contrast, there are additional factors that need to be considered, i.e. the position of the measurements on the platform plays a major role. As shown in 3.3, the transmission angle through the f-theta lens has a significant influence on both the measurement of absolute temperature and the uncertainty calculation. The influence of the angle-dependent transmission properties of the f-theta lens on the total measurement uncertainty is shown in Fig. 9.

As with most attempts to measure absolute temperatures in additive manufacturing, the emissivity of the material is probably the most critical parameter [26]. However, the SPIT-approach provides a unique advantage by enabling measurements in an area located outside the process zone, where the material has already solidified completely. This method allows the determination of the emissivity of the material under investigation in advance. Based on the separate emissivity measurements it is thus possible to consider the anticipated surface properties and the relevant temperature range. The surface condition during the in-process measurements may in some situations deviate from that of the sample body for the determination of the emissivity and thus lead to deviations in the measured absolute temperature. Nevertheless, the procedure shown here provides the possibility to determine absolute temperatures in large parts of the process with high accuracy.

Since the basic calibration was performed on a blackbody, it is valid for any material in the PBF-LB/M. It is only necessary to perform the independent emissivity measurement of the material with the corresponding surface properties in the desired temperature range.

## 5 Conclusion

The paper presents a calibration method for an infrared thermography system with a mobile measurement field which is guided through a complex optical path for the use in PBF-LB/M. The method employs a two-sided blackbody cavity in form of a graphite tube and relies on the comparison of the grey values of the infrared detector with reference temperatures. By using a multi-point method with up to six interpolation points, the resulting temperatures agree well with the reference temperatures and include the wavelength-dependent transmission properties of the optical path over the desired temperature range. Due to the complexity of the optical path and its irregular transmission properties, a pixel-by-pixel calibration is used. To ensure a broad application in PBF-LB/M, several configurations with different optical paths, sensor exposure areas and integration times were developed. The setup and the recording parameters are optimized to cover two different temperature ranges with high frame rates. One temperature range near the melting point of stainless steel 1.4540 for process monitoring during the build job, and the other in a lower temperature region for subsequent active thermography investigations. The investigation of the influence of the position on the platform and, therefore, the transmission angle of radiation through the f-theta lens demonstrates a reduction of the signal by approximately 2.5% at maximum deflection.

The presented calibration method provides the ability to measure absolute temperatures during the processing in PBF-LB/M. Furthermore, it provides measurement parameters for investigations in different temperature ranges with high temporal resolution. Thus, different aspects and characteristics of the manufacturing process can be investigated in detail.

## Funding

This research was done as part of the ViPAF project funded by 10.13039/501100005017StMWI - Bayern Innovativ Bayerische Gesellschaft für Innovation und Wissenstransfer mbH, grant number 41–6562b/25/2-VAL-2103-0006.

## Data availability statement

The data presented in this study has not been deposited into a publicly available repository but is available on request from the corresponding author.

## CRediT authorship contribution statement

**Dennis Höfflin:** Writing – review & editing, Writing – original draft, Visualization, Validation, Software, Methodology, Investigation, Funding acquisition, Formal analysis, Data curation, Conceptualization. **Christian Sauer:** Writing – review & editing, Writing – original draft, Visualization, Validation, Software, Methodology, Investigation, Formal analysis, Data curation, Conceptualization. **Andreas Schiffler:** Writing – review & editing, Supervision, Software, Resources, Project administration, Methodology, Funding acquisition, Conceptualization. **Jochen Manara:** Writing – review & editing, Investigation. **Jürgen Hartmann:** Writing – review & editing, Supervision, Software, Resources, Project administration, Methodology, Funding acquisition, Conceptualization.

## Declaration of competing interest

The authors declare that they have no known competing financial interests or personal relationships that could have appeared to influence the work reported in this paper.

## Nomenclature

$a_{n}$: Interpolation gradient
A,B,C: Curve fitting parameter
$b_{n}$: Interpolation initial value
$c_{1},c_{2}$: Radiation constants
d: Distance camera to object
G: Geometric factor of the camera system
$I_{\text{photo}}$: Photo signal of the camera system sensor
l: Length of the graphite cavity
$L_{\text{bb}}$: Spectral infrared radiation of a blackbody
$L_{\text{real}}$: Spectral infrared radiation of a real surface
r: Radius of the graphite cavity
s(λ): Spectral sensitivity of the camera system sensor
*t*_*n*,95_: Student's distribution
$T_{a}$: Ambient temperature
$T_{\text{atm}}$: Atmospheric temperature
$T_{\text{bb}}$: Blackbody temperature
$T_{\text{bb},\text{cam}}$: Blackbody temperature, measured by the camera system
$T_{\text{bb},\text{cam}}$: Blackbody temperature, measured by pyrometer
$T_{\text{cavity}}$: Temperature of the cavity
$T_{\text{process}}$: Process temperature
$T_{\text{real}}$: Temperature of a real surface
$u_{\text{pyro}}$: Partial uncertainty of the pyrometer
$u_{r,\bar{T}}$: Mean random error
$u_{T_{\text{bb},\text{cam}}}$: Partial uncertainty of the blackbody temperature, measured by the camera system
$u_{T_{\text{bb},\text{pyro}}}$: Partial uncertainty of the blackbody temperature, measured by pyrometer
$u_{\text{temp}}$: Overall uncertainty budget
$u_{{\Delta T}_{\text{Cavity}}}$: Partial uncertainty of the Temperature of the cavities
$u_{\varepsilon_{\text{cavity}}}$: Partial uncertainty of the emissivity of the graphite cavity
$u_{\varphi_{\alpha }}$: Partial uncertainty of angular deviation correction factor
V: Standard deviation
α: Transmission angle
ε: Emissivity of a surface
$\varepsilon_{\text{cavity}}$: Emissivity of the graphite cavity
$\varepsilon_{\text{graphite}}$: Emissivity of the graphite surface
λ: Wavelength
$\lambda_{\text{eff}}$: Effective narrowband wavelength
$\lambda_{\min}$: Minimum wavelength of the optical path
$\lambda_{\max}$: Maximum wavelength of the optical path
$\varphi_{\alpha }$: Angular deviation correction factor
ω: Relative humidity

These methods are used to assess process stability within defined limits, as they have limited interpretability due to the lack of information on fundamental thermal physics. To gain comprehensive and insightful process information in LPF-LB/M and to understand the fundamental physics that causes the temperature-related process phenomena and changes in component properties, in-situ measurement of transient absolute temperatures is mandatory.

There is only a limited number of technologies and methods for monitoring and measuring the temperature throughout the ultrafast process necessary for capturing the rapid melting and cooling of the material. A major challenge here is estimating the correct emissivity of the material, which varies with temperature and state of matter and can hardly be determined in the process zone.

The two-wavelength approach offers a way to measure temperature detached from the problem of unknown emissivity in the process zone by simultaneously measuring at two different, closely spaced wavelengths. The method measures temperature by determining the ratio of intensities of two wavelength bands of emitted electromagnetic radiation, assuming the same emissivity at both wavelengths. This approach utilizes the fact that the intensity ratio changes with temperature in a known and predictable manner to calculate the temperature of the object under examination. In order to develop a layer-wise melt pool control, temperature measurements at a single location in the melt pool have been achieved with the application of two-color pyrometry to PBF-LB/M [6]. Although pyrometry can provide good temporal resolution of the region of interest, it is limited to a single location and therefore cannot determine spatial temperature distributions. Misalignment of the optical setup used to gather thermal radiation of the melt pool can result in inaccurate temperature values. Insufficient resolution can lead to incorrect calculated temperature values as the radiation is collected from areas with non-uniform temperature distribution. An approach yielding both, high spatial and temporal resolution of the melt pool has been presented by Hooper [11] using coaxial imaging with high-speed cameras. A two-wavelength imaging setup was used to account for emissivity changes. Temperatures in the approximate range of 1000 K–4000 K were measured. Recording at 100 kHz with a resolution of 20 μm made it possible to record thermal gradients in the range of 5 K/μm - 20 K/μm and cooling rates in the range of 1 K/μs - 40 K/μs.

Common other approaches to measure absolute temperatures requiring only one measuring device utilize a setup including an infrared camera system. For this method, a calibration of the camera plays a key role and is usually carried out via blackbody radiators. The material's emissivity is a crucial parameter here, while the process zone's emissivity estimation poses significant challenges.

Deisenroth et al. utilize a hemispherical reflectometer and the establishment of a relationship between reflectance and emissivity in their research to provide a sophisticated approach to emissivity measurement [12]. The study reports varying emissivity depending on the state and history of the surface of high-purity nickel samples. On the virgin surface, an emissivity of approximately 0.39 was measured. This value decreased to values between 0.36 and 0.37 near the solidification temperature. The lowest emissivity values are observed in the molten and solidified material behind the tail, with typical values in the range of 0.31–0.35. This is attributed to increased reflectance as the solid liquid transition has eliminated the grinding marks of the original surface.

Further calibration approaches use fundamental physical constants to establish a correlation between the measured temperature radiation and the actual temperature. Lane et al. [13] used the temperature plateau of resolidification in a setup with a combination of a CMOS and an InSb camera to determine the emissivity of IN625 at the known solidification temperature after calibrating with the use of a black body radiator. To obtain temperatures during the cooling process of stainless steel 316L, Scheuschner et al. [14] also used the solidification temperature of the material to perform a single-point calibration. By applying Planck's law and considering the previously determined intensity values they scaled the result linearly to correlate the thermal radiation with the absolute temperature values. This method provides the benefit of considering every element of the optical path in the calibration process. Nevertheless, the precision of these measurements is constrained by not accounting for additional factors, including wavelength-dependent characteristics of the optical elements, as well as the impact of the observation angle and a detailed consideration of the material emissivity. A multi-point method can overcome many challenges of a single-point calibration. Mohr et al. [15] employed thermocouples to calibrate an infrared system for estimating the emissivity of powder layers and bulk material in laser powder bed fusion. This method yielded multiple reliable reference points to correlate the infrared radiation with. Due to the temperature durability of the thermocouples, measurements were performed to a maximum of 900 K. The calibration accounted for the elements of the optical path, as it was performed in the intended measurement setup. The emissivity was then derived from the temperatures measured by the IR camera and the thermocouple, considering the manufacturer-provided absorption properties of the mirrors and glasses. Thus, the emissivity estimation and the entire calibration depend on the accuracy of the provided data and neglect the temperature-dependent changes in the absorption properties of the optical elements. Dinwiddie et al. [16] developed a method to measure the thermal radiation properties of materials used in electron beam melting (EBM). A test specimen with three features was designed: a cylindrical cavity to simulate a blackbody cavity, an as-printed surface to represent the reduced radiation of a real surface, and a sintered powder pocket to estimate the emittance of powder material. The specimen was heated up to 1500 K and an infrared camera was calibrated using four thermocouples embedded in the specimen. This allows to simultaneously determine both the absorption of the optical path and the emissivity of the solidified material over temperature.

To overcome the difficulties of the highly dynamic material emissivity in the process zone and to record absolute temperatures during the PBF-LB/M process, the Synchronized Path Infrared Thermography (SPIT) approach [5] looks promising. Here, the measuring field of a high-speed infrared camera, controlled via a separate scan head, tracks the laser-material interaction zone at a defined distance. In this way, absolute process temperatures can be determined in the already solidified material with a known emissivity and used for further investigations. To obtain accurate temperature measurements with a high-speed infrared camera, proper calibration is still essential. Factors such as the considered wavelength range, the transmission properties of the optical path as well as sensor-specific sensitivity properties influence the accuracy of the measurement results significantly.

This paper presents the calibration process of an infrared imaging device in the SPIT configuration to achieve accurate results for the laser powder bed fusion process. This involves the characterization of the optical path, relevant data collection and the determination of optimal hardware and software parameters. The curve-fitting process is performed in a two-step approach based on the physical principles as well as a correction of the specific attenuation properties of the optical path.
